# Supplementary material for: Evidence for igneous differentiation in Sudbury Igneous Complex and impact-driven evolution of terrestrial planet proto-crusts
Source: Nat Commun. 2019 Jan 31;10:508. doi: 10.1038/s41467-019-08467-9 (PMC6355857; doi:10.1038/s41467-019-08467-9)
Supplement: Supplementary file 1 — Description of Additional Supplementary Files [file 41467_2019_8467_MOESM1_ESM.pdf]

Supplementary Data 1: Data 1: Major and trace element data for rocks from the Elm street and Creighton traverses, South Range of the Sudbury Igneous Complex, Canada (for Fig. 5; 7a, c, d)

Supplementary Data 2. Data 2. Sm-Nd isotopic analyses for blocks of mafic norite from the Elm street and Creighton traverses, South Range of the Sudbury Igneous Complex, Canada (for Fig. 7c)
